# Effect of Solvents on Fe–Lignin Precursors for Production Graphene-Based Nanostructures

**DOI:** 10.3390/molecules25092167

**Published:** 2020-05-06

**Authors:** Qiangu Yan, Zhiyong Cai

**Affiliations:** 1 Ligwood LLC, Madison, WI 53705-2828, USA; yanqiangu@gmail.com; 2 Forest Products Lab, USDA Forest Service, Madison, WI 53726-2398, USA

**Keywords:** kraft lignin, Fe–lignin precursors, graphene-based nanomaterials, solvents, water, methanol, acetone, tetrahydrofuran (THF)

## Abstract

Kraft lignin was catalytically graphitized to graphene-based nanostructures at high temperature under non-oxidative atmospheres. To obtain the best catalytic performance, a uniform catalyst–lignin mixture must be made by bonding transitional metal (M) ions to oxygen (O), sulfur (S) or nitrogen (N)-containing functional groups in kraft lignin. One of the strategies is to dissolve or disperse kraft lignin in a suitable solvent, whereby the polymer chains in the condensed lignin molecules will be detangled and stretched out while the functional groups are solvated, and when mixing lignin solution with catalyst metal solution, the solvated metal ions in an aqueous solution can diffuse and migrate onto lignin chains to form M-O, M-S, or M-N bonds during the mixing process. Therefore, solvent effects are important in preparing M–lignin mixture for production of graphene-based nanostructures. Fe–lignin precursors were prepared by dissolving lignin with different solvents, including water, methanol, acetone, and tetrahydrofuran (THF). Solvent effects on the catalytic performance, size and morphology of graphene-based nanostructures were investigated using X-ray diffraction (XRD), field emission scanning electron microscopy (FE-SEM), high resolution transmission electron microscopy (HRTEM), and nitrogen sorption measurements. The sizes, morphologies, and catalytic properties of the products obtained from Fe–lignin precursors are greatly influenced by the solvents used. It was found that Fe–lignin (THF) had the highest iron dispersion and the smallest iron particle size. Furthermore, Fe–lignin (THF) exhibited the best catalytic performance for graphitization of kraft lignin while the graphitization degree decreased in the order: Fe–lignin(THF) > Fe–lignin(Acetone) > Fe–lignin(methanol) > Fe–lignin(water).

## 1. Introduction

To massively manufacture low-priced graphene materials, a molecular cracking and welding (MCW) method has been developed for producing few-layer graphene from solid carbon resources, especially biomass sources [1,2]: graphene-encapsulated transitional metal nanostructures are first produced by graphitization of mixtures of metal catalyst and solid carbon materials. Then, graphene-encapsulated transitional metal structures are cracked by ‘scissoring molecules’, and the cracked few-layer graphene shells are peeled off the metal cores to serve as building block units for self-welding and reconstructed into high-quality, few-layer graphene materials under the heating temperature with selected welding reagent gases [1,2]. According to the MCW concept, graphene-encapsulated core–shell nanoparticles are first prepared by catalytic graphitization of solid carbon resources [1,3]. To obtain good catalytic effect, a uniform catalyst-solid carbon mixture must be made [2,4]. A major issue in catalytic graphitization of solid carbon resources including biomass is the contact degree between catalysts and raw carbon materials [5]. Therefore, a uniform distribution of metal catalysts in solid carbon matrix is the prerequisite. Any solid carbon source can be used for production of graphene-encapsulated core–shell nanoparticles, and Kraft lignin is used as the carbon precursor to demonstrate the production of few-layer graphene through the MCW method [1]. Poor dispersion of metal particles on lignin and larger iron particle sizes was found unfavorable to increase its contact degree with lignin, which can significantly lower the graphitization activity to lignin [2]. The degree of catalyst–solid carbon contact is generally poor if a metallic catalyst powder is just simply mixed with lignin [6]. Practically, metal salts, instead of metal particle powder, are used to obtain high metal dispersion in solid carbon and improve its catalytic performance [2,4]. Catalyst–solid biomass material precursors are usually prepared with an impregnation method: a dry biomass material is added to a metal salt solution which will be absorbed by the biomass material because of the capillary forces produced by its inner pores. Metal ions diffused into a biomass matrix can be anchored in the biomass through bonding to biomass oxygen-containing groups [7]. However, unlike lignosulfonates which are highly water soluble due to the presence of the sulfonic acid groups, kraft lignin has a limited solubility in most solvents, especially water. Lignin in general is a 3-D, chemically cross-linked phenolic polymer. Specifically, kraft lignin has relatively high hydrophobicity since it is condensed to huge molecules when recovered from black liquors through the acidity precipitation process; the polymer chains are entangled and curled, and a metal salt aqueous solution is repelled due to its hydrophobic property. Therefore, it is very difficult for metal ions to penetrate kraft lignin particles if an impregnation method is used, which yields a poor contact degree between metal and kraft lignin [2,4]. Fortunately, there are significant oxygen-containing functional groups of lignin, i.e., aliphatic hydroxyl in the propane sidechains, phenolic hydroxyl, methoxyl, and carbonyl groups [8]. There are also nitrogen- and sulfur-bonded functional groups like −N-H (amide), and −S-H (sulfide) [9]. Metal ions will react and bond with these functional groups if these functional groups are approachable to metal ions. One of the strategies is to dissolve or disperse kraft lignin in a suitable solvent, allowing the polymer chains in the condensed lignin molecules to be detangled and stretched out in the solvents, while the functional groups are solvated and can be reached by metal ions. Significant research work has been done on lignin solubility and swelling in different solvents [10,11] through isolating lignin from pulping processes, purification of lignin and modifying lignin structures to improve its physical-chemical properties for different applications. Schuerch [10] first examined lignin solubility in more than 30 different solvents, and found the solubility increasing in the following order: water < benzene < methanol < ether < acetone < pyridine. Saito et al. [11] studied the fractionation of softwood kraft lignin using methanol as the solvent. Jiang et al. [12] isolated the softwood kraft lignin into four different portions by sequential precipitation with various organic solvents. Studies demonstrate the solubility of lignin in organic solvents is affected by many variables such as chemical structure, molecular weight, and the presence of hydrophilic moieties in the lignin molecule [13]. Chemical modification of lignin structure such as acetylation can improve the solubility of lignin in organic solvents [14,15]. The dissolution behavior of lignin depends on its isolating/extraction process: lignosulfonate is soluble in water, kraft and soda lignins are soluble in alkali solutions, while organosolve lignin is soluble in most of organic solvents [16]. Kraft lignin is usually isolated from black liquors by precipitation through acidification method during kraft pulping process. Its solubility increases with a decrease of molecular weight, due to the higher disentanglement degree [14]. Different organic solvent systems have been investigated to dissolve kraft lignin [14,17], such as alcohols [14,17], carboxylic acids [18,19], ketones [14,20], esters [14,17], ethers [14,17], and heterocyclic compound like pyridines, dioxane and tetrahydrofuran [14,18,19]. 

A ‘co-precipitation’ technique [2,4] was developed to prepare catalyst–lignin mixture to produce graphene nanostructures from kraft lignin, i.e., both kraft lignin and metal salts were first pre-dissolved in a solvent separately. Metal (M) salts are dissolved in de-ionized (DI) water, while kraft lignin is dissolved or dispersed in organic solvents. Then, both solutions and/or suspensions are well mixed, and metal salt and lignin precipitate as solid particles. Metal ions are uniformly anchored over functional groups of lignin chains during mixing and precipitating process. The M–lignin precursors are obtained after vaporizing the solvents. To prepare a uniform M–lignin mixture, it is very important to select a suitable solvent to dissolve or disperse kraft lignin, and the selected solvent should be miscible with water since the catalyst metal salt is usually dissolved in an aqueous solution. In the present work, methanol, acetone and tetrahydrofuran are selected for dissolution kraft lignin. Iron is the catalyst, and iron (III) nitrate has previously been proved the most efficient iron catalyst [4], and is therefore used as the iron source in this work. The effect of the solvents, including water, methanol, acetone, and tetrahydrofuran (THF) on the depolymerization of lignin will be investigated. The solvent effects on the distribution of iron catalyst in lignin will be evaluated to determine bonding forms of Fe metal ion and lignin in different solution systems before pre-heat treatment and investigate effects of different solvents on the uniformity of Fe ion dispersion in lignin.

## 2. Experimental

### 2.1. Materials

The kraft lignin used in this study as a carbon source was BioChoice lignin provided by Domtar Inc. (Plymouth, NC, USA), which is a pine kraft lignin produced at its Plymouth mill by the LignoBoost process. The received wet lignin sample (~25–30 wt% moisture) was first naturally dried in air for three weeks, the moisture content was then decreased to about 7.0 wt%, and this sample is denoted as raw kraft lignin. All chemicals and solvents used were of certified ACS reagent grade and purchased from Sigma-Aldrich, Inc. (St. Louis, MO, USA). The solvents, methanol, acetone, and tetrahydrofuran (THF) were used as received.

**Figure 1:**
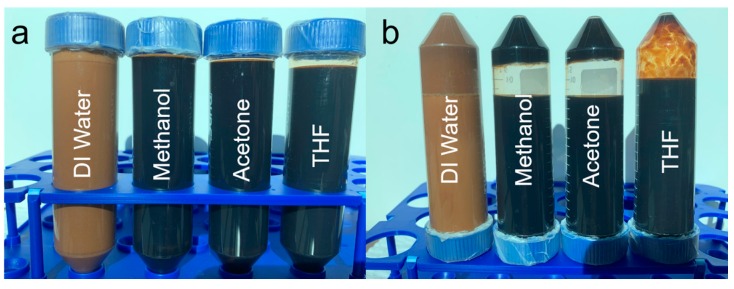
Photos of lignin–water, lignin–methanol, lignin–acetone, and lignin–THF mixtures after being agitated over an orbital shaker overnight at room temperature (**a**), then settled for 30 min and turned upside down (**b**).

The interaction between kraft lignin and different solvents were performed by dissolving or dispersion of each 10 g raw lignin sample in 50 mL de-ionized (DI) water, methanol, acetone, and THF, respectively; each lignin–solvent mixture was agitated over an orbital shaker overnight at room temperature. Figure 1 showed photos of the four immediately well mixed samples (Figure 1a) and the four samples were left to stand for 30 min and then turn upside down (Figure 1b). It was observed that the well mixed lignin–water mixture was in a light brown suspension condition, and almost all the lignin precipitated from water after settling for 30 min. The lignin–methanol, lignin–acetone, and lignin–THF seemed to form dark solutions after shaking for overnight (Figure 1a); however, about 40% of lignin deposited from methanol after standing for 30 min, and roughly 30% lignin precipitated from acetone and nearly no lignin separated from THF solution after standing for 30 min. The four mixture was placed in a fume hood until the solvent was completely vaporized and the sample was finally dried in the fume hood at room temperature for 2 weeks. The dried lignin samples were denoted as lignin–M (methanol), lignin–A (acetone), and lignin–T (THF). The moisture content in the raw lignin, lignin–M, lignin–A, and lignin–T samples was measured by following ASTM D4442-07 standard [21].

### 2.2. Preparation of Fe–Lignin Mixture

Previous experimental results showed that the lignin graphitization degree and conversion level to graphene materials increased significantly with the increase of iron/lignin mass ratio, and the optimized Fe/lignin ratio was 1/9 [22]. Iron–lignin precursors with a mass ratio of 1/9 (1 part iron and 9 parts lignin) were prepared at room temperature using a co-precipitation technique: 300 g of raw lignin was first added to each of four different solvents (500 mL each), i.e., de-ionized (DI) water, methanol, acetone, and THF in a 2000 mL glass beaker, respectively. In addition, the mixture was stirred for 2 h to obtain the lignin–solvent blend. An amount of 246.0 g of iron (III) nitrate nonahydrate (Sigma-Aldrich) was added to 100 mL DI water in a 500 mL glass beaker, followed by stirring the mixture until iron nitrate was dissolved completely. The iron nitrate solution was added to the lignin–DI water mixture and the iron nitrate–lignin–solvent mixture was stirred for 2 h. The final mixture was kept at room temperature for 24 h and then oven-dried at 110 °C for 24 h. After the drying process, a solid iron–lignin mixture was obtained as a precursor. The same mixing and drying procedures were performed for the other three lignin–solvent mixtures: lignin–methanol, lignin–acetone, and lignin–THF. The Fe–lignin precursors from the four different solvents are denoted as Fe–lignin–W (water), Fe–lignin–M (methanol), Fe–lignin–A (acetone), and Fe–lignin–T (THF).

### 2.3. Catalytic Graphitization

The catalytic graphitization process has been described in the previous work [4]. The details are described as follows: 15 g of an Fe–lignin composite was packed in the middle of a 1-inch OD ceramic tubular reactor. High-purity argon (99.99% purity) gas was first introduced into the reactor at a flow rate of 50 mL/min for 30 min. The reactor was heated at a rate of 10 °C/min to 1000 °C and kept at 1000 °C for 1 h. The furnace was cooled down naturally to room temperature. 

### 2.4. Thermogravimetric Analyses (TGA) 

TGA of iron–lignin samples were conducted using a TGA (Shimadzu TGA-50H, Marlborough, MA, USA) [4]. For each run, 10 mg of samples were loaded with argon (99.99% purity, 50 mL/min) gas flowing through the TGA at 50 mL/min as temperature was ramped at 10 °C/min. Each of the samples was repeated for three times. 

### 2.5. Formation of Gaseous Products by Temperature-programmed Decomposition (TPD)

TPD of raw kraft lignin and Fe–lignin precursors were performed using a fix-bed flow system, and the gaseous products generated during the TPD process was quantitatively analyzed by an on-line Hiden QGA quantitative gas analysis system [4]. The signals from the mass spectra of 2, 15, 28, 31, 34, 44, 78 and 94 (*m/z*) were identified as major contributors from evolved gases and volatiles (H_2_, CH_4_, CO, CH_3_OH, H_2_S, CO_2_, benzene, and phenol).

### 2.6. Characterization 

The FTIR spectra of the raw lignin, lignin–M, lignin–A, lignin–T and Fe–lignin samples were recorded with the PerkinElmer attenuated total reflection (ATR) spectrometer (PerkinElmer, Waltham, MA, USA) at a resolution of 2 cm^−^^1^ for 40 scans in 450 to 4000 cm^−^^1^ range to determine structural changes of kraft lignin after mixing with different solvents and iron solution. The XRD (X-ray Diffraction) patterns were recorded on a Bruker D8 Advance diffractometer at 40 kV and 44 mA using Cu-Kα radiation with a wavelength of 1.5406 Å, from 15° to 80° at a scan rate of 0.02° s^−1^. The surface areas of solid samples were determined by a Quantachrome, Autosorb-1 through the Brunauer–Emmett–Teller (BET) gas adsorption method. Prior to measurements, all samples were degassed at 300 °C for 3 h. The morphology of samples was investigated using a Field Emission Scanned Electron Microscope (FESEM). All SEM samples were pre-coated with 10 nm Pt before introduced into the vacuum chamber. The system was operated with an accelerating voltage of 5 kV. Transmission Electron Microscope (TEM) images were obtained using a JEOL JEM-2100 operated at 200 kV. All TEM samples were sonicated in ethanol solution for 1 min before transfer to carbon grids. 

## 3. Results and Discussion

### 3.1. FTIR

**Figure 2:**
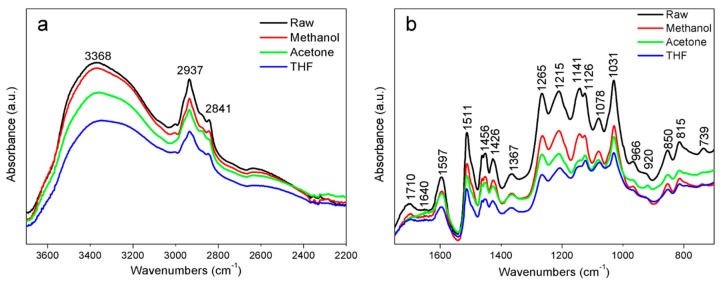
FTIR spectra of raw KL, and lignin–W, lignin–M, lignin–A, and lignin–T at the ranges of 3800 to 2200 cm^−1^ (**a**) and 1750 to 700 cm^−1^ (**b**).

Infrared spectra of the kraft lignin samples treated with different solvents were compared to spectra of the raw lignin (Figure 2). The raw lignin sample exhibited a strong broad peak at 3368 cm^−1^ (Figure 2a), which is attributed to the stretching vibrations of hydroxyl groups in phenolic and aliphatic side chains. The stronger hydroxyl peak might also have been partially contributed by the moisture trapped in tangled lignin chains [23]. Kraft lignin is recovered from black liquors through the acidity precipitation process, where the condensed lignin chains are twisted and entangled, forming a huge 3-D structure, and a portion of water molecules are held and trapped in the lignin particles during its isolating/extraction process. The moisture content in kraft lignin sample was measured and the results were reported as 6.8 ± 0.5%, 3.5 ± 0.3%, 1.8 ± 0.2% and 0.5 ± 0.2% for raw lignin, lignin–M, lignin–A, and lignin–T samples, respectively. The intensities of the absorption peak at 3368 cm^−1^ decreased in the order of raw lignin < lignin–M < lignin–A < lignin–T, where the decreasing absorption peak agreed with the order of moisture content. This result can be elucidated by the solubilities of kraft lignin in different solvents. According to previous reports [14], the solubility of kraft lignin in solvents used in current work is ordered as: THF>Acetone>Methanol>Water. When dissolving kraft lignin in a suitable solvent, polymer chains in kraft lignin are detangled and uncurled, huge lignin molecules become accessible to solvent molecules, and part of moisture trapped in condensed lignin particles may be extracted into the solvent. There are also a range of signs around 2937 (C-H stretchings in aromatic methoxyl groups as well as in methyl and methylene groups of side chains) and 2841 cm^−1^ (symmetric C-H stretching in -CH2-and tertiary C-H groups) typically found in lignin. There is no change observed in these bands for samples after being treated with different solvents. 

Figure 2b shows a comparison of the absorption bands of 800–1750 cm^−1^ between the raw lignin and three samples treated with methanol, acetone and THF solvent. Only minor differences were observed between these samples meaning no significant changes to functional groups of kraft lignin after dissolving in solvents. The absorption bands were strongly consistent with assigned band of chemical components as mentioned in previous literature [24,25]. In the carbonyl region, weak to medium bands are found at 1710 cm^–1^ that can be associated with unconjugated C=O, a shoulder absorption peak at 1640 cm^–1^ related to ring conjugated C=C stretch of coniferyl/sinapyl alcohol spectra, and the sharp peak at 1597 cm^−1^ corresponds to vibrations in the aromatic ring symmetric stretching. A very sharp absorption at approximately 1511 cm^−1^ corresponds to aryl ring asymmetric stretching vibrations for softwood lignin (Guaiacyl-G). The medium band at 1456 cm^−1^ is assigned to the asymmetric deformation of C-H bonds, while the band at 1426 cm^−1^ corresponds to the vibration of aromatic rings of lignin. The band at 1367 cm^−1^ represents the contribution of OH bending to non-etherified phenolic -OH groups. A strong absorption band at 1265 cm^−1^ is assigned to C-O vibration of guaiacyl rings of softwood kraft lignin, while the C–O stretching vibration in syringol rings is designated to the peak at 1215 cm^−1^. The band at 1141 cm^−1^ is due to guiacyl C-H and syringyl C-H/aromatic C-H on-plane deformation in the guaiacyl ring of the softwood kraft lignin. A band at 1126 cm^−1^ is related to aromatic C-H in-plane deformation in guaiacyl rings, guiacyl C-H and syringyl C-H/aromatic C-H on-plane deformation in the guaiacyl ring in hardwood kraft lignin. A 1078 cm^−1^ band is assigned to C-O deformations of secondary alcohols and aliphatic ethers. The very sharp absorption peak at 1031 cm^−1^ is assigned to aromatic C-H in plane deformation (G > S). 

**Figure 3:**
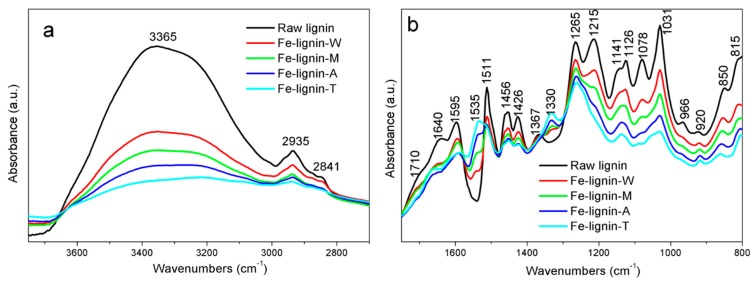
FTIR spectra of KL, and Fe–lignin–W, Fe–lignin–M, Fe–lignin–A, and Fe–lignin–T at the ranges of 3750 to 2700 cm^−1^ (**a**) and 1750 to 800 cm^−1^ (**b**).

FTIR spectra of kraft lignin and Fe–lignin samples prepared with four different solvents are compared in Figure 3. It was found that the significant changes in intensity and shift in position of the peaks were due to introduction of iron nitrate. The first observed change was the attenuation of the intensity and shift of the peaks in the region of 3700–3300 cm^−1^ and 1100–900 cm^−1^, indicating a decrease of the free hydroxyl group on the kraft lignin. The decrease of the intensity and shift of the peaks 1710, 1640, 1458, 1367, 1215, and 1078 cm^−1^ suggests interaction between iron ions and the related C=O, and C-O groups. These changes may be explained by iron ions associated with carboxylate and hydroxylate anions, revealing that carbonyl, carboxyl and hydroxyl groups are the main active sites to anchoring Fe^3+^ [26]. There were also a couple of new bands at 1535 and 1330 cm^−1^ showing the presence of surface nitrate complexes from iron nitrate [27,28,29,30]. Decrease in absorption intensity of hydroxyl, C=O, and C-O related groups for Fe–lignin samples is ordered Fe–lignin–T> Fe–lignin–A> Fe–lignin–M> Fe–lignin–W, while the intensities of nitrate complex peaks increase with the order of Fe–lignin–W < Fe–lignin–M < Fe–lignin–A < Fe–lignin–T. 

### 3.2. Thermal Analyses of Fe–Lignin Precursors Prepared Using Different Solvents

**Figure 4:**
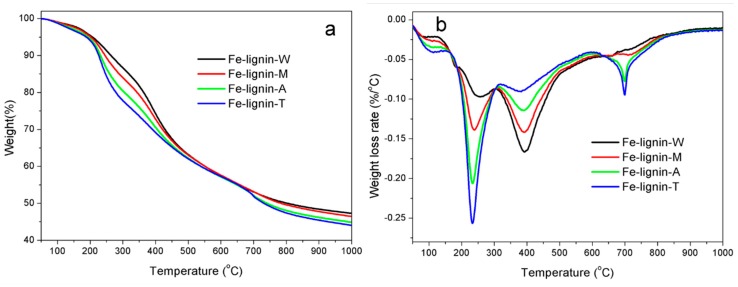
TG (**a**) and DTG (**b**) curves of kraft lignin and Fe–lignin prepared with four different solvents: DI water, methanol, acetone and THF.

FTIR spectra demonstrated that there were many oxygen-containing (carboxylic, carbonyl, acyl, alkoxyl, ketones, esters, and ethers, et al.), alkyl, and aromatic functional groups in kraft lignin [31]. These functional groups in kraft lignin were broken down or cracked with the increase in temperature during the thermal process. All samples were characterized by TGA under an argon atmosphere (Figure 4) to demonstrate the effects of solvents on thermal properties of kraft lignin and Fe–lignin samples. Figure 4 shows TG and DTG curves of raw lignin and four Fe–lignin precursors, i.e., Fe–lignin–W, Fe–lignin–M, Fe–lignin–A, and Fe–lignin–T. Figure 4a shows that solid residues as percentage of starting weights of iron-promoted lignin precursors after decomposition were 47.3%, 46.5%, 44.9%, and 44.0% for Fe–lignin–W, Fe–lignin–M, Fe–lignin–A, and Fe–lignin–T, respectively, which indicates that the catalytic graphitization activity of four iron catalysts on kraft lignin materials was in ascending order Fe–lignin–W < Fe–lignin–M < Fe–lignin–A < Fe–lignin–T. The thermal decomposition process of Fe–lignin precursors can be divided into four stages (Figure 4b). 

The first stage is characterized by a mass loss due to evaporation of surface moisture and dehydration of combined moistures from iron-promoted lignin precursors. The peak temperatures of Fe–lignin samples in the first stage were 106.8, 106.9, 113.7, and 116.5 °C for Fe–lignin–W, Fe–lignin–M, Fe–lignin–A, and Fe–lignin–T precursors, respectively; and the peak mass loss rates were 0.02168, 0.02764, 0.03496, and 0.04094% °C^−1^ for the four Fe–lignin samples prepared with water, methanol, acetone, and THF, respectively.

The second stage corresponded to the catalytic decomposition of kraft lignin and iron compounds. During the decomposition process, the functional groups in alkyl side chains of lignin basic units were catalytically decomposed [2,4]. The mass decreased rapidly due to the breakage of large number of ether and C-C bonds connected on phenyl propane units, which generated gaseous products and condensable volatiles [2,4]. The maximum rates of these weight losses occurred at the temperatures of 254.7 °C, 240.0 °C, 232.5 °C and 231.9 °C for Fe–lignin–W, Fe–lignin–M, Fe–lignin–A, and Fe–lignin–T precursors, respectively. The peak mass loss rates were 0.09697, 0.1389, 0.2062, and 0.2566% °C^−1^ for Fe–lignin–W, Fe–lignin–M, Fe–lignin–A, and Fe–lignin–T precursors, respectively.

The third mass loss (Figure 4), corresponding to the catalytic decomposition of solid residue yielded from the second stage, indicated that the remaining functional groups of kraft lignin continued to decompose as the temperature increased, which led to the formation of char matrix. The peak temperatures in the third stage were 392.5, 290.0, 387.9, and 380.0 °C for Fe–lignin–W, Fe–lignin–M, Fe–lignin–A, and Fe–lignin–T precursors, respectively, showing peak temperature decreasing for the four Fe–lignin samples prepared with water, methanol, acetone, and THF, respectively. The weight loss of this stage, decreasing in the order of Fe–lignin–W > Fe–lignin–M > Fe–lignin–A > Fe–lignin–T, suggests the functional groups left in the solid residues from the second stage are in the same order. The peak loss rates were 0.1666, 0.1417, 0.1142, and 0.0905% °C^−1^ for Fe–lignin–W, Fe–lignin–M, Fe–lignin–A, and Fe–lignin–T precursors, respectively.

The fourth mass loss stage was characterized with a further carbonization and graphitization process of lignin chars in a wider temperature range up to 1000 °C, where the mass loss was mainly because of oxygen elimination in carbonyl and quinone groups, and hydrogen in C-H groups of lignin chars, which freed as CO and H_2_. The peak temperatures in this stage were 720, 710, 700, and 699 °C for Fe–lignin–W, Fe–lignin–M, Fe–lignin–A, and Fe–lignin–T samples, respectively. The peak loss rates were 0.0379, 0.0.0443, 0.0774, and 0.0948% °C^−1^ for Fe–lignin–W, Fe–lignin–M, Fe–lignin–A, and Fe–lignin–T precursors, respectively.

### 3.3. Gaseous Product Analyses by Temperature-Programmed Decomposition (TPD)

**Figure 5:**
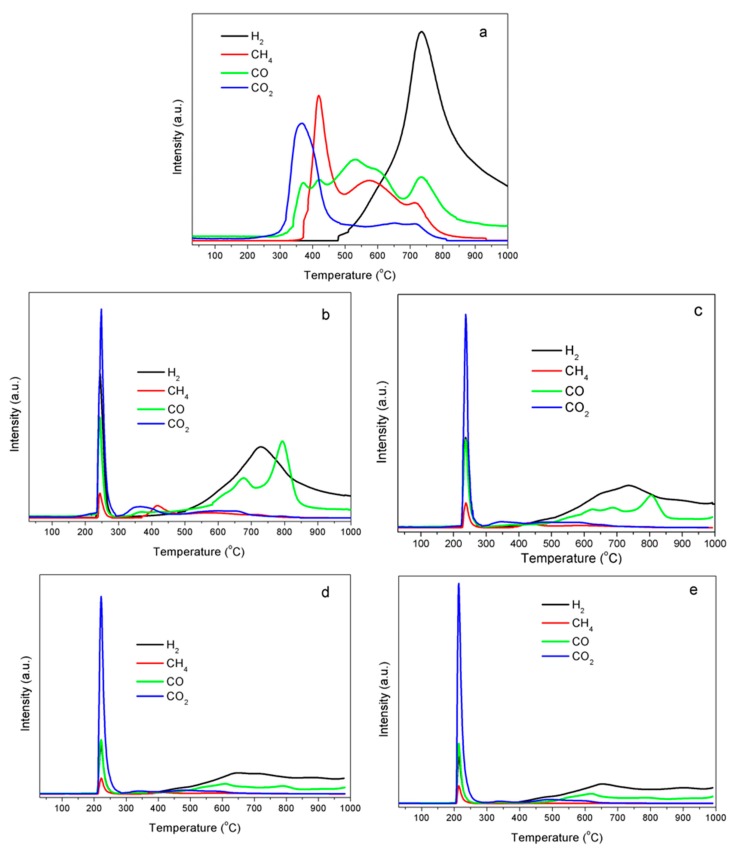
Evolution of gas products CO_2_, CO, H_2_ and CH_4_ from thermally treated Fe–lignin samples prepared with different solvents: (**a**) raw lignin, (**b**) Fe–lignin–W, (**c**) Fe–lignin–M, (**d**) Fe–lignin–A, and (**e**) Fe–lignin–T.

The basic structure of lignin is based on the phenyl propanoid unit, consisting of an aromatic ring and a 3-C side chain; and the huge lignin molecule is cross-linked by three main types of phenylpropane: 4-hydroxy-3-methoxyphenylpropane (coumaryl alcohol), 3,5-dimethoxy-4-hydroxyphenylpropane (sinapyl alcohol), and 4-hydroxyphenylpropane (coniferyl alcohol) [32]. Lignin can be gradually bio-degraded by fungi and bacterial organisms with the aid of catalysts, oxidative agents, or thermal treatment, where lignin is chemically degraded by breaking down at 1, 4, 5, and β positions [29], accompanying the formation of incondensable gases H_2_, CO_2_, CO; light hydrocarbons, CH_4_, C_2_H_6_, C_2_H_4_; organic volatiles, CH_3_OH, HCHO, C_6_H_6_OH; water vapor, and sulfur-containing products, H_2_S, SO_2_, and CS_2_ [33]. Figure 5 shows the evolution curves of release intensities of the main gaseous components (CO_2_, CO, H_2_ and CH_4_) during the temperature-programmed decomposition of raw kraft lignin and Fe–lignin samples. Gaseous products were mainly released over 300 °C for the raw lignin sample (Figure 5a). Two major CO_2_ peaks were detected during the decomposition of kraft lignin under an argon atmosphere (Figure 5a). CO_2_ released during lignin decomposition is attributed to the decomposition of carboxyl (-COO^−^) and ester (-CO-O-R) groups of kraft lignin [22,34]. Carboxyl was mainly responsible for the low temperature peak of 367 °C. The CO_2_ evolution at high temperature 653 °C was assigned to ester groups when the thermal process was under an inert atmosphere. The CO formation is mainly contributed by decomposition of carbonyl, ether, phenol and quinone groups in raw lignin and the intermediate char products [33]. At least four CO evolution peaks were observed for raw kraft lignin samples under an argon atmosphere. The CO evolution peaks at low temperature measured 369 and 421 °C, which were mainly attributed to decomposition of the weakly bonded ether (α-O-4 and β-O-4) bridges and decarbonylation of the carbon on the C3 side-chain [34,35,36]. The CO evolution peak at 530 °C was attributed to the cleavage of aromatic bonded oxygens (i.e., methoxy and phenolic groups), and the CO peak at 734 °C was assigned to quinone groups in the char from pyrolysis of lignin [7] and the secondary cracking of oxygenate volatiles and tars [37]. Methane was released from the decomposition of kraft lignin between 370 and 800 °C under an argon atmosphere and was mainly contributed by the cracking of methoxy groups, decomposition of side aliphatic chains, the rearrangement of lignin carbon skeleton during carbonization process and the secondary pyrolysis of aromatic volatile intermediates (Figure 5a). The CH_4_ evolution peaks at 418 °C were mainly contributed by the breaking down of weakly bonded methoxy groups (O-CH3) [34,35,38] and the fragmentation of side aliphatic chains [35]. The evolution of CH_4_ peak at 575 °C was assigned to the rearrangement of lignin carbon skeleton along with CO release to remove the residual oxygen [34,35], and the methane peak at 717 °C might be contributed by the secondary pyrolysis of aromatic volatile intermediates [39,40]. The thermal decomposition of kraft lignin in Ar atmosphere eliminates the hydrogen portion via thermal cracking of C−H in aliphatic CHx (x = 1~3) and aromatic rings [7,41]. The hydrogen formation temperature under Ar atmosphere (Figure 5a) started at 477 °C, reached its maximum at 739 °C, and continued to over 1000 °C.

Gas formation trends of Fe–lignin samples are significantly different compared to raw lignin (Figure 5b–e). Gaseous products were released within two temperature zones during the heating process and at temperatures between 150 and 300 °C, lignin was catalytic depolymerized by iron ions at this stage. The second stage was the catalytic carbonization/graphitization process occurring in the temperature range of 300–1000 °C. In the process of depolymerization, cracking occurs with the admixture catalyst. With the assistance of iron species, there is a cleavage of the lignin macromolecular matrix to stable, low molecular weight, liquid and gaseous products, as well as a solid char residue. Lignin depolymerization is a complex process consisting of many reactions: decarboxylation, decarbonylation, dehydration, cracking, isomerization, condensation, aromatization, and pyrolysis, gaseous and volatile products, H_2_O, CO_2_, CO, H_2_, CH_3_OH, C_6_H_5_OH, HCHO, CH_4_ and other light hydrocarbons are formed through these reactions [33]. The depolymerization temperature of lignin is lowered by adding iron catalysts. In this work, the depolymerization temperatures of the Fe–lignin samples are: 249 °C for the Fe–lignin–W (Figure 5b), 237 °C for the Fe–lignin–M (Figure 5c), 222 °C for the Fe–lignin–A (Figure 5d), and 213 °C for the Fe–lignin–T (Figure 5e), respectively. The lower depolymerization temperature illustrates better catalytic performance for the Fe–lignin–T sample, due to the high solubility of lignin in THF, which makes the best iron distribution in the lignin macromolecule matrix of the four solvents used in this work. The gases formed in this stage are mainly CO_2_, H_2_, CO, and CH_4_, and the relative amount of each product is in the order of CO_2_>H_2_ ≅ CO >CH_4_ for all the four Fe–lignin samples, and the total amount of the gases produced in this stage is in the order of Fe–lignin–T> Fe–lignin–A> Fe–lignin–M> Fe–lignin–W. In general, a higher catalyst–lignin contact degree led to an increase in gas production during lignin catalytic decomposition process [22], and the high levels of gaseous products formed in this process show that THF was the best solvent for this work.

In the catalytic graphitization process, the solid char formed in the first stage is further heated and the rest of heteroatoms (O, H, N and S) are eliminated from the carbon matrix as the gaseous products, H_2_O, CO_2_, CO, H_2_, etc. [33,42]. It is observed that less gaseous products are released from Fe–lignin samples compared to that of raw lignin (Figure 5), because most of the heteroatoms are removed in the catalytic depolymerization process stage. While the total gas amount produced in this stage is ordered Fe–lignin–T< Fe–lignin–A<Fe–lignin–M< Fe–lignin–W, it is contrary to the order of the catalytic depolymerization step. This further proves THF is a better solvent to prepare an Fe–lignin precursor with high iron dispersion.

### 3.4. Effects of Solvents on Fe–Lignin Performance—Product Distributions

Table 1 summarizes the solid (dry basis excluding iron), liquid, and gas product distributions of Fe–lignin samples indicating that in general, catalytic performance of the thermal treatment process was in the order of Fe–lignin–T > Fe–lignin–A > Fe–lignin–M > Fe–lignin–W. All these were evidenced by the fact that raw kraft lignin had a solid carbon yield of 36.5%, while Fe–lignin samples yielded 34.3%, 33.1%, 31.4%, and 30.8% for Fe–lignin–W, Fe–lignin–M, Fe–lignin–A, and Fe–lignin–T, respectively. Usually, a higher iron dispersion led to an increase in gas production, therefore, a higher carbonization degree since more heteroatoms were removed from the char matrix by catalytic action [22].

### 3.5. Solid Product Characterization

#### 3.5.1. Surface Area

The Brunauer–Emmett–Teller (BET) surface areas of solid products from raw kraft lignin and Fe–lignin samples are listed in Table 2. The results indicate the BET surface areas are 15.3, 59.1, 85.3, 110.6 and 129.2 m^2^/g for raw lignin, Fe–lignin–W, Fe–lignin–M, Fe–lignin–A, and Fe–lignin–T, respectively. During catalytic carbonization process, active metal iron played a direct role in breaking down the bonds between carbon and heteroatoms and the formation of physical structures of solid residues [22]. A better iron distribution in the lignin char matrix might result in the formation of a higher surface area of the solid product. The BET surface area of Fe–lignin samples prepared with different solvents was in the order of Fe–lignin–T > Fe–lignin–A > Fe–lignin–M > Fe–lignin–W in this work. This could be explained by the importance of solvent in preparing high contact degree of Fe–lignin composite: (1) Kraft lignin has a high solubility in THF, where polymer chains in the condensed lignin molecules will be detangled and stretched out when dissolved in THF, while the functional groups are solvated and can be accessed by the metal ions. When the lignin–THF solution and the iron nitrate solution are well mixed, Fe^3+^ ions are uniformly anchored over functional groups of lignin chains during mixing process, iron salt and lignin are co-precipitated as Fe–lignin solid particles. (2) In contrast, kraft lignin is almost insoluble in DI water, a suspension will be formed when mixing lignin powder and water, the polymer chains in lignin remain condensed and tangled due to the hydrophobic property of kraft lignin. When mixing the lignin–water suspension and iron nitrate solution, Fe^3+^ ions will be repelled from the lignin particles and will not reach over and be chelated by the functional groups [2,4,22], iron salt then gathers up over lignin surface, which leading to poor contact degree between iron and lignin during the coprecipitation. During catalytic graphitization process, iron species in the Fe–lignin–W sample tend to agglomerate and merge into larger sized particles. This fact could cause sintering of iron particles and growth of iron particles, and thus, poor catalytic performance of the sample and a decrease of the solid residue surface area [4].

#### 3.5.2. X-ray Diffraction (XRD)

**Figure 6:**
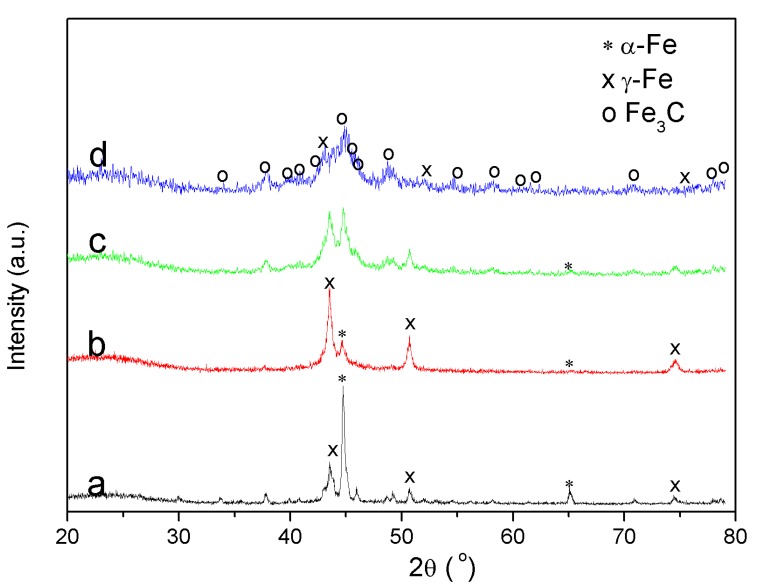
XRD patterns of thermal treated Fe–lignin samples prepared using different solvents, Fe–lignin–W (**a**), Fe–lignin–M (**b**), Fe–lignin–A (**c**), and Fe–lignin–T (**d**).

In the current study, the structure and crystallographic planes of the catalytically graphitized products of Fe–lignin samples prepared with different solvents were analyzed by XRD. Figure 6 shows the XRD patterns of graphitized Fe–lignin samples prepared using different solvents. Mainly, iron species were characterized by XRD in the samples, i.e., metallic α-Fe (JCPDS, No. 87-0722), γ-Fe (JCPDS, No. 89-3939), and Fe_3_C (JCPDS, No. 89–2867) in Figure 5, respectively. The graphitized Fe–lignin–W and Fe–lignin–M samples exhibited sharp and narrow peaks of metallic iron (mainly α-Fe and γ-Fe). Catalytically graphitized Fe–lignin–A sample showed the metallic iron peaks (α-Fe and γ-Fe) and small and broad peaks of iron carbide (Fe_3_C). Graphitized Fe–lignin–T sample showed weak and broad peaks of metallic iron (mainly γ-Fe) and iron carbide (Fe_3_C), which indicated the presence of smaller Fe particles in the Fe–lignin–T sample compared to the Fe–lignin–W and Fe–lignin–M samples. The weak-broad diffraction peaks observed in the Fe–lignin–T sample suggested good dispersion of iron species in the product. The sharper diffraction peaks of the Fe–lignin–W and Fe–lignin–M samples implied a growth in the crystallite size of metallic irons or iron carbide (Fe_3_C) and poor iron dispersion in the lignin matrix. The mean particle size was calculated for the most intense diffraction peaks of α-Fe, γ-Fe and Fe_3_C using the Scherrer equation [4]. Only α-Fe and γ-Fe were detected in the Fe–lignin–W and Fe–lignin–M samples, while α-Fe, γ-Fe, and Fe3C were observed in Fe–lignin–A and Fe–lignin–T samples. The mean sizes of α-Fe nanoparticles were 78.7, 42.3, 18.5.1, and 5.5 nm for Fe–lignin–W, Fe–lignin–M, Fe–lignin–A and Fe–lignin–T samples, respectively; while the γ-Fe grain sizes averaged 17.4, 25.1, 11.8, and 7.9 nm, respectively. The mean sizes of Fe_3_C nanoparticles were 12.7 and 7.8 nm for Fe–lignin–A and Fe–lignin–T samples, respectively. These results indicate that a suitable solvent can make uniformly dispersed smaller iron nanoparticles that are beneficial for obtaining a high performing iron catalyst toward graphitization to kraft lignin, while a poor solvent will make a poor mixed Fe–lignin precursor leading to sintered, larger iron nanoparticles and worsening the catalytic performance of irons as a catalyst [4].

#### 3.5.3. Scanning Electron Microscope (SEM)

**Figure 7:**
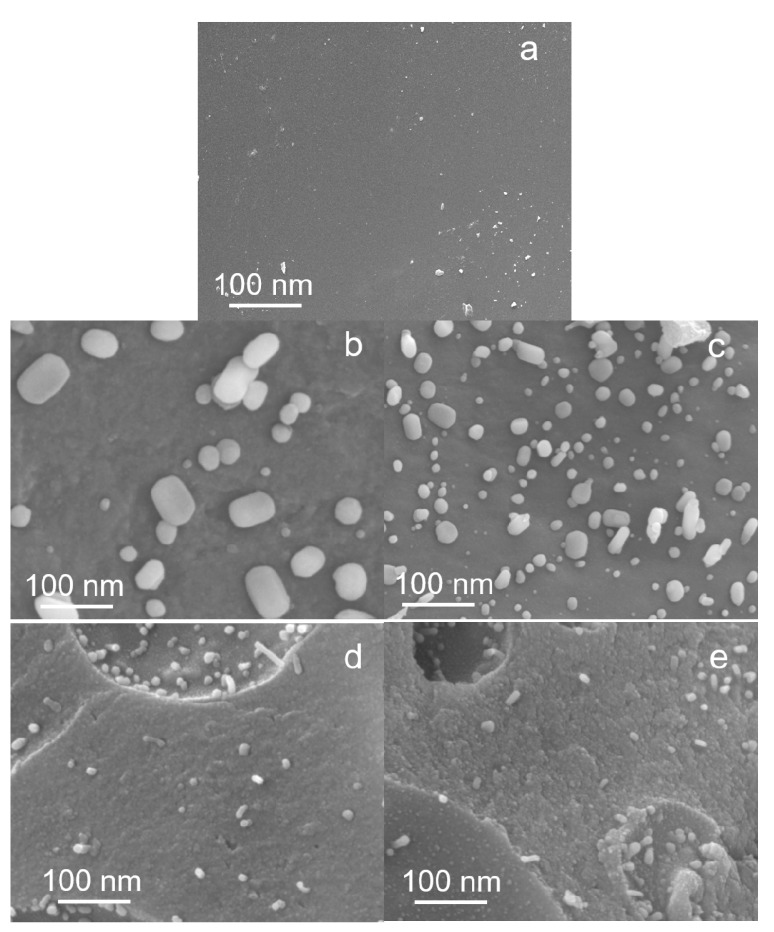
Scanning electron microscope images of raw lignin and four Fe–lignin samples thermally treated at 1000 °C for 1 h under an argon flow: raw lignin (**a**), Fe–lignin–W (**b**), Fe–lignin–M (**c**), Fe–lignin–A (**d**) and Fe–lignin–T (**e**).

The SEM images (Figure 7) show typical morphologies observed on thermally treated Fe–lignin samples prepared with four different solvents of Fe–lignin–W, Fe–lignin–M, Fe–lignin–A and Fe–lignin–T. The morphologies of Fe–lignin–W, and Fe–lignin–M samples were significantly different from those of Fe–lignin–A and Fe–lignin–T samples.

Scanning electron microscopy (SEM) was used to characterize the morphology of the thermal treated raw lignin and Fe–lignin samples. Figure 7 shows the SEM morphologies of the raw kraft lignin and the Fe–lignin prepared using different solvents. The surface of raw kraft lignin thermally decomposed under argon flow (Figure 7a) was smoother and cleaner. In the cases of water and methanol solvents, the large iron particles observed on the surface of the thermal treated Fe–lignin–W and Fe–lignin–M are attributable to the poor solubility of kraft lignin in water and methanol. Specifically, the diameter of the surface iron particles using water solvent was about 80–100 nm (Figure 7b). The particles were smaller when methanol was used as the solvent, but their diameter ranged between 20–50 nm. In the case of the acetone solvent, fewer particles were observed on the surface, the iron particles were well distributed in the lignin–derived carbon matrix, and the spherical iron particles were smaller, their diameter being only about 10–20 nm. The morphology of the iron particles when THF was used as the solvent was even smaller with a relatively uniform particle size ranging between 5 to 10 nm, these spherical particles were uniformly distributed in the carbon matrix after heat treatment. XRD results proved that these nanoparticles were composed of α-Fe, γ-Fe, iron carbide, and graphene structures.

#### 3.5.4. High-Resolution Transmission Electron Microscopy (HRTEM)

**Figure 8:**
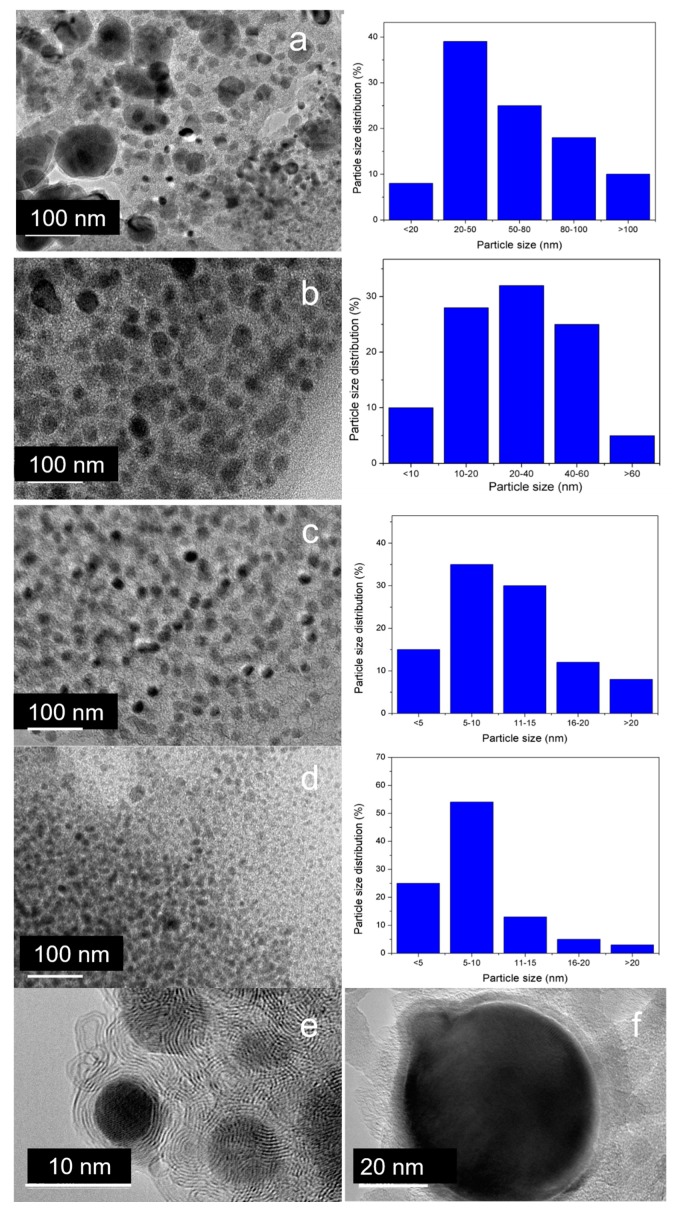
High-resolution transmission electron microscope images of four Fe–lignin precursors Fe–lignin–W (**a**), Fe–lignin–M (**b**), Fe–lignin–A (**c**), and (**d**) Fe–lignin–T after thermal-treated at 1000 °C for 1 h under an argon flow, high-magnification HRTEM images of Fe–lignin–T (**e**), and Fe–lignin–W (**f**).

Figure 8 shows HRTEM images and iron particle size distribution of graphitized Fe–lignin samples prepared using different solvents. The black particles in HRTEM images were iron nanoparticles scattered in the lignin carbon residue. The nanoparticles in the Fe–lignin–T (Figure 8d,e) sample had core–shell structures with diameter of core nanospheres in the 3–10 nm range. The carbon shells displayed ordered graphene plane structures (Figure 8e). These core(Fe)–shell(graphene) nanoparticles were evenly embedded in the amorphous carbon matrices [4]. The morphology of Fe–lignin–W (Figure 8a) and Fe–lignin–M (Figure 8b) samples was much different from the Fe–lignin–T sample. When water and methanol were used as solvent, serious agglomeration of iron particles occurred, and the particle size grown as large as 50–100 nm in diameter. Most of the iron-core nanoparticles in Fe–lignin–W, and Fe–lignin–M samples were shelled with disordered amorphous carbon structures (Figure 8f) and were unevenly distributed in the amorphous carbon matrix. Iron nanoparticles in Fe–lignin–W, and Fe–lignin–M samples were agglomerated to large sizes, which was in agreement with the XRD results. HRTEM and XRD results revealed that uniformly dispersed iron nanoparticles were beneficial for achieving a more efficient catalyst to the graphitization of kraft lignin, while sintered larger iron nanoparticles deteriorated the catalytic performance of irons as a catalyst [22]. Crystallite size of iron nanoparticles prepared from different solvents was observed in the order of Fe–lignin–W > Fe–lignin–M > Fe–lignin–A > Fe–lignin–T.

## 4. Conclusions

In the present study, a series of Fe–lignin precursors were synthesized using different solvents including DI water, methanol, acetone and THF for catalytic graphitization to graphene-based nanostructures. Among four Fe–lignin precursors, Fe–lignin–T had the highest iron dispersion and the smallest particle size. It is considered that the solvents, which are used to dissolve kraft lignin, remarkably influence the interaction between iron ions and the functional groups on lignin. The carbonyl, carboxyl and hydroxyl groups will bond with iron ions to form Fe–lignin chelation which anchors iron species and prohibits the sintering of iron nanoparticles during the catalytic graphitization process. These results show that THF is the best solvent among those considered for the preparation of graphene-based nanostructures from kraft lignin. However, THF is considered to be a toxic solvent, so searching for a non-toxic solvent is one of the future tasks for this work. To use black liquor directly as the lignin resource is probably another option to eliminate the organic solvent from the catalytic graphitization lignin to graphene materials.

## Figures and Tables

**Table 1:** Product weight percentage distribution of catalytic decomposition of Fe–lignin precursors prepared with different solvents.

Fe Resources	Solid Carbon	Liquid	Gas
Raw lignin	36.5	24.2	39.3
Fe–lignin–W	34.3	19.6	46.1
Fe–lignin–M	33.1	19.0	47.9
Fe–lignin–A	31.4	17.5	51.1
Fe–lignin–T	30.8	17.5	51.7

**Table 2:** Surface area (S_g_) of carbon-based nano materials from kraft lignin prepared with different solvents.

Samples	S_g_ (m^2^ g^−1^)
Raw lignin	15.8 ± 2.5
Fe–lignin–W	59.1 ± 4.6
Fe–lignin–M	85.3 ± 3.5
Fe–lignin–A	110.6 ± 5.0
Fe–lignin–T	129.2 ± 2.3
